# Molecular prevalence and diagnostic gene targets of Anaplasma, Babesia, Theileria, and Trypanosoma in cattle and buffaloes across Southeast Asia (2015–2025): A systematic review and meta-analysis

**DOI:** 10.14202/vetworld.2026.4054-4068

**Published:** 2026-09-14

**Authors:** Nitipon Srionrod, Tran Nhat Thang, Pornchai Pornpanom, Noppason Pangprasit

**Affiliations:** 1 Faculty of Veterinary Medicine, Mahanakorn University of Technology, Bangkok 10530, Thailand.; 2 Faculty of Animal Science and Veterinary Medicine, Thai Nguyen University of Agriculture and Forestry, Thai Nguyen 24119, Vietnam.; 3 Akkhraratchakumari Veterinary College, Walailak University, Nakhon Si Thammarat 80160, Thailand.; 4 One Health Research Center, Walailak University, Nakhon Si Thammarat 80160, Thailand.; 5 Informatics Innovation Center of Excellence, Walailak University, Nakhon Si Thammarat 80160, Thailand.

**Keywords:** Anaplasma, Babesia, cattle, meta-analysis, molecular epidemiology, polymerase chain reaction, Southeast Asia, Theileria

## Abstract

**Background and Aim:**

Hemoparasitic infections caused by *Anaplasma*, *Babesia*, *Theileria*, and *Trypanosoma* adversely affect cattle and buffalo health, resulting in economic losses and reduced livestock productivity in Southeast Asia. Although numerous molecular epidemiological studies have been conducted in individual countries, a comprehensive regional synthesis of polymerase chain reaction (PCR)-confirmed prevalence and molecular diagnostic targets has been lacking. This study aimed to estimate the pooled molecular prevalence of major hemoparasites in cattle and buffaloes across Southeast Asia, summarize the PCR target genes used for their detection, and investigate the effects of geographical region and host species on prevalence.

**Materials and Methods:**

A systematic review and meta-analysis were conducted following the Preferred Reporting Items for Systematic Reviews and Meta-Analyses guidelines. PubMed, ScienceDirect, and Scopus were searched for studies published between 2015 and December 31, 2025. Cross-sectional studies conducted in Southeast Asia that used PCR-based methods and reported extractable prevalence data were included. Pooled prevalence estimates were calculated using random-effects meta-analysis. Heterogeneity was assessed using Cochran's Q test and the I² statistic, whereas meta-regression was performed to evaluate the influence of geographical region (mainland versus maritime Southeast Asia) and host species. Publication bias was assessed for *Babesia* spp.

**Results:**

Twenty-two cross-sectional studies involving 5,789 cattle and 901 buffaloes met the inclusion criteria. The pooled prevalence was 12% (95% confidence interval [CI]: 3%-38%) for *Anaplasma* spp., 18% (95% CI: 11%-28%) for *Babesia* spp., 11% (95% CI: 3%-35%) for *Theileria* spp., and 11% (95% CI: 4%-28%) for *Trypanosoma evansi*. Species-specific pooled prevalences were 5% for *Anaplasma marginale*, 10% for *Babesia bigemina*, 9% for *Babesia bovis*, and 10% for *Theileria orientalis*. Significant between-study heterogeneity was observed across all analyses. Meta-regression identified a significantly higher prevalence of *B. bovis* in maritime than mainland Southeast Asia (p = 0.0048), whereas no significant regional or host species effects were detected for the remaining hemoparasites. The review also compiled the principal PCR target genes used for molecular diagnosis, including *16S rRNA*, *groEL*, *MSP4*, *RAP-1a*, *AMA-1*, *SBP-2*, *SBP-4*, *MSA*, *MPSP*, *RoTat* 1.2 *VSG*, *ITS1*, *ITS2*, and satellite DNA.

**Conclusion:**

Major bovine hemoparasites remain widely distributed across Southeast Asia, highlighting the need for strengthened molecular surveillance and standardized diagnostic approaches. This study provides the first comprehensive regional synthesis of PCR-confirmed prevalence and molecular diagnostic targets for major hemoparasites in cattle and buffaloes, offering valuable evidence to support veterinary diagnostics, surveillance programs, and disease control strategies based on a One Health approach.

## INTRODUCTION

The agricultural sector is an important contributor to the economy of Southeast Asia accounting for approximately 11%–22% of the gross domestic product (GDP) of Southeast Asian countries in 2022 [1]. In some countries, such as Thailand and Vietnam, the agriculture, forestry, and fishing sector grew by 3.6% and 3.4%, respectively, in 2025 [2, 3]. In recent years, global population growth and economic development have accelerated the demand for red meat, particularly in Southeast Asian countries, making food security a continuing regional priority [4]. In Thailand, large ruminants (cattle, dairy cattle, and buffaloes) are among the five most important livestock species contributing to national livestock production [5]. Beyond commercial dairy and beef production systems, indigenous cattle are widely raised in rural communities as a source of household income and financial security. Similarly, livestock production is an integral component of rural livelihoods in Vietnam [6] and plays an important role in agricultural sustainability.

Ticks are important arthropod vectors of pathogens affecting both veterinary medicine and public health in Southeast Asian countries, where diverse geographical and ecological conditions support a rich diversity of tick species [7, 8]. Tick-borne pathogens include viruses, bacteria, protozoa, and helminths [7], among which *Anaplasma*, *Babesia*, haemotropic *Mycoplasma*, and *Theileria* are major pathogens affecting livestock health [8, 9]. *Anaplasma marginale* causes anemia in cattle through erythrophagocytosis by splenic macrophages following infection of erythrocytes, resulting in anemia without hemoglobinemia or hemoglobinuria [10]. In contrast, *Babesia* spp. invade and replicate within erythrocytes, causing direct erythrocyte destruction that leads to anemia, jaundice, hemoglobinuria, and fever [11]. *Theileria orientalis* infection is generally considered subclinical or asymptomatic [12]. However, cases of fever, hemolytic anemia, and mortality have been reported in Australia and New Zealand [13].

Another important blood-borne pathogen of cattle and buffaloes is *Trypanosoma evansi*, a salivarian trypanosome that causes surra [14]. This protozoan parasite is mechanically transmitted by tabanid and *Stomoxys* flies, of which 128 and 14 species, respectively, have been reported in Thailand [15]. Because the regional environment favors arthropod vector development and vector species are highly abundant [8, 15], the transmission of economically important hemoparasites remains a persistent challenge in Southeast Asian countries. Therefore, updated information on the molecular prevalence and diagnostic detection of these pathogens is needed to strengthen disease surveillance, improve veterinary diagnostic capacity, and support effective management of large ruminant health. Although a previous meta-analysis evaluated zoonotic tick-borne pathogens in Southeast Asian countries [16], and another systematic review focused on trypanosomes in Indonesia [17], no previous study has synthesized polymerase chain reaction (PCR)-confirmed prevalence data for the four major hemoparasite genera (*Anaplasma*, *Babesia*, *Theileria*, and *Trypanosoma*) in cattle and buffaloes across Southeast Asia during 2015–2025. Furthermore, no previous review has compared molecular prevalence between mainland and maritime Southeast Asian countries.

Despite increasing reports on bovine hemoparasites from individual Southeast Asian countries, comprehensive regional evidence integrating PCR-confirmed prevalence, molecular diagnostic targets, and sources of epidemiological heterogeneity remains unavailable. Consequently, regional differences in hemoparasite distribution and the molecular markers used for diagnosis have not been systematically evaluated, limiting evidence-based surveillance and diagnostic standardization across Southeast Asian countries.

Therefore, this systematic review and meta-analysis aimed to (i) estimate the pooled molecular prevalence of *Anaplasma marginale*, *Babesia bigemina*, *Babesia bovis*, *Theileria orientalis*, and *Trypanosoma evansi* in cattle and buffaloes across Southeast Asian countries; (ii) summarize the molecular diagnostic gene targets used for the detection of major hemoparasites during the past decade; (iii) investigate mainland–maritime and host species heterogeneity using meta-regression analyses; and (iv) provide evidence-based reference data to support veterinary diagnostic laboratories, disease surveillance, and hemoparasite control programs in Southeast Asia.

## MATERIALS AND METHODS

### Ethical approval

This systematic review and meta-analysis was conducted in accordance with the Preferred Reporting Items for Systematic Reviews and Meta-Analyses (PRISMA) guidelines [18].

### Study period and location

The literature search, study selection, data extraction, data analysis, and interpretation of the findings were conducted from January 2026 to April 2026 at Akkhraratchakumari Veterinary College, Walailak University, Nakhon Si Thammarat, Thailand.

### Protocol registration, database searching, and article screening

The review protocol was registered with the Open Science Framework (OSF; DOI: 10.17605/OSF.IO/BFWQR). Relevant articles reporting the prevalence of hemoparasites in cattle and buffaloes (*Anaplasma*, *Babesia*, *Theileria*, *Trypanosoma*, and *Mycoplasma*) were retrieved from three bibliographic databases: PubMed, ScienceDirect, and Scopus. The search strategy included the following search terms: ("vector-borne diseases" OR "tick-borne diseases") AND ("cattle" OR "buffaloes"); ("*Anaplasma*" OR "*Babesia*" OR "*Theileria*" OR "*Trypanosoma*") AND ("cattle" OR "buffaloes"); and ("prevalence" OR "occurrence"). Articles published between 2015 and December 31, 2025, were eligible for inclusion. Three reviewers (NP, NS, and PP) independently screened titles and abstracts.

### Study selection and eligibility criteria

Studies were included in the systematic review if they met the following criteria: (i) cross-sectional study design; (ii) target population comprising cattle, buffaloes, or both; (iii) conducted in Southeast Asia; (iv) sample size ≥25 [19]; (v) use of PCR-based detection methods; and (vi) reporting the exact numbers of examined and positive animals or prevalence with a 95% confidence interval (CI). Studies were excluded if they (i) were not relevant to the prevalence of hemoparasites; (ii) investigated animal species other than cattle or buffaloes; (iii) used non-PCR-based detection methods; (iv) were case reports or review articles; (v) provided insufficient data for effect-size calculation; (vi) included fewer than 25 animals; (vii) were conducted outside Southeast Asia; or (viii) contained duplicate data published elsewhere. Full-text assessment and final study selection were independently performed by two reviewers (NP and NS).

### Quality assessment and data extraction

Two reviewers (NS and TNT) independently extracted data, and discrepancies were resolved through discussion with a third reviewer (PP). The extracted information included (i) study characteristics (authors and publication year); (ii) methodological characteristics (sampling location, study period, and parasite detection method); (iii) host characteristics (host species, age, sex, total number of animals examined, and number of positive animals); and (iv) parasite characteristics (parasite species). The methodological quality of the included studies was independently assessed by two reviewers (NS and PP), with disagreements resolved through discussion. The quality assessment criteria were adapted from a previously published study [19]. Studies with quality scores of 0–8 were classified as low quality, whereas those with scores of 9–14 were classified as high quality.

### Data synthesis and data analysis

Quantitative data, including the total numbers of cattle and buffaloes examined and the numbers infected with *Anaplasma marginale*, *Anaplasma centrale*, *Anaplasma bovis*, *Anaplasma platys*, *Babesia bigemina*, *Babesia bovis*, *Babesia ovata*, *Theileria orientalis*, *Theileria annulata*, *Theileria sinensis*, *Theileria* sp., *Trypanosoma evansi*, *Mycoplasma wenyonii*, and *Candidatus Mycoplasma haemobos*, were extracted and pooled for analysis. Pooled prevalence estimates were calculated for parasite groups represented by at least three eligible studies. Eight pooled prevalence analyses were performed, including overall estimates for *Anaplasma* spp., *Babesia* spp., and *Theileria* spp. infecting cattle and buffaloes. Species-specific pooled prevalence estimates were also calculated for *A. marginale*, *B. bigemina*, *B. bovis*, *T. orientalis*, and *Tr. evansi*. In addition to estimating pooled prevalence, this study systematically summarized the PCR gene targets used by laboratories across Southeast Asian countries for the molecular detection of major hemoparasites during 2015–2025.

A meta-analysis of proportions was performed using a random-effects model in R (version 4.5.3) [20] according to established methodological guidelines [21]. Between-study heterogeneity was assessed using Cochran's Q test and the I² statistic. Low heterogeneity was defined as p ≥ 0.10 or I² <50%, whereas high heterogeneity was defined as p ≤ 0.10 or I² >50% [22]. Forest plots were generated to visualize pooled prevalence estimates. Where substantial heterogeneity was detected, meta-regression analyses were performed to evaluate differences in the prevalence of *A. marginale*, *B. bigemina*, *B. bovis*, *T. orientalis*, and *Tr. evansi* according to geographical region (mainland vs. maritime Southeast Asian countries) and host species (cattle vs. buffaloes). Publication bias was assessed using funnel plots and Egger's regression test only for *Babesia* spp., because fewer than ten studies were available for the remaining parasite groups [23].

## RESULTS

### Characteristics of included studies

Database searching initially identified 4,803 articles (Figure 1). After removing 1,572 duplicate records, 3,157 articles were excluded during title and abstract screening because they did not meet the eligibility criteria. The full texts of 74 articles were subsequently assessed for eligibility, of which 51 were excluded for the following reasons: 12 were not relevant to the study objectives [24–35], seven provided insufficient data for effect-size calculation [36–42], ten used detection methods outside the scope of this review [43–52], one included a sample size of fewer than 25 animals [53], one was not a cross-sectional study [54], two were review articles, systematic reviews, or meta-analyses [55, 56], and 18 were conducted outside Southeast Asia [57–74].

**Figure 1:**
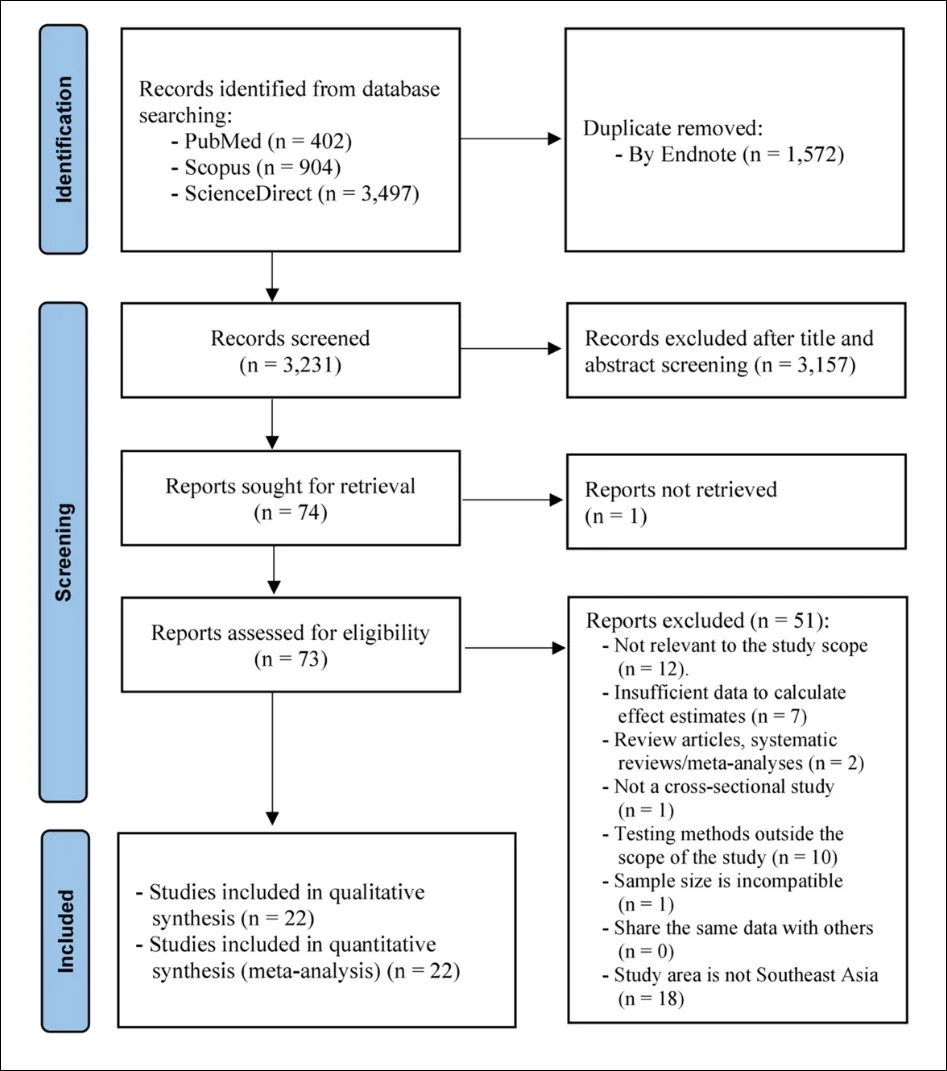
Preferred Reporting Items for Systematic Reviews and Meta-Analyses (PRISMA) flow diagram illustrating the identification, screening, eligibility assessment, and inclusion of studies in the systematic review.

A total of 22 cross-sectional studies were included in the qualitative and quantitative analyses, and their characteristics are summarized in Supplementary File 1. Among these studies, 14 investigated hemoparasites in both beef and dairy cattle [9, 75–87], three investigated buffaloes only [88–90], and five included both cattle and buffaloes [91–95]. *Anaplasma* spp. were reported in nine studies [9, 75, 76, 80–83, 85, 88], *Babesia* spp. in ten studies [77–81, 84, 87, 88, 90, 95], *Theileria* spp. in seven studies [9, 79–81, 88, 90, 92], *Trypanosoma evansi* in five studies [9, 86, 89, 91, 93], and *Trypanosoma theileri* in one study [91]. Two other pathogens, *Candidatus Mycoplasma haemobos* [9] and *Mycoplasma wenyonii* [94], were also reported.

Four *Anaplasma* species were identified, namely *Anaplasma centrale* [82], *Anaplasma bovis* [85], *Anaplasma marginale* [9, 75, 76, 80–83, 85, 88], and *Anaplasma platys* [9, 76, 85]. Three *Babesia* species were reported, including *Babesia bigemina* [77–81, 84, 87, 88, 90], *Babesia bovis* [77–81, 87, 88, 90, 95], and *Babesia ovata* [88]. Likewise, three *Theileria* species were identified, namely *Theileria orientalis* [9, 79–81, 88, 90], *Theileria sinensis* [9], and *Theileria* sp. [92]. These hemoparasites were reported from Indonesia [78, 86], Malaysia [9, 77, 82–84], the Philippines [88, 89, 91, 94], Thailand [75, 79–81, 85, 87, 90, 92, 93], and Vietnam [76, 95]. Quality assessment demonstrated that all 22 included studies were of high methodological quality (Figure 2).

Among the included studies, nine reported hemoparasite co-infections [9, 78–82, 88, 90, 91]. Three studies described co-infections in buffaloes, including dual infections [88, 90, 91] and triple infections [90]. The reported dual co-infection patterns included *A. marginale* with *B. bigemina*, *A. marginale* with *B. bovis*, *B. bigemina* with *B. bovis*, *B. bigemina* with *T. orientalis*, *B. bovis* with *T. orientalis*, and *Tr. evansi* with *Tr. theileri*. The only reported triple co-infection in buffaloes comprised *B. bigemina*, *B. bovis*, and *T. orientalis*.

**Figure 2:**
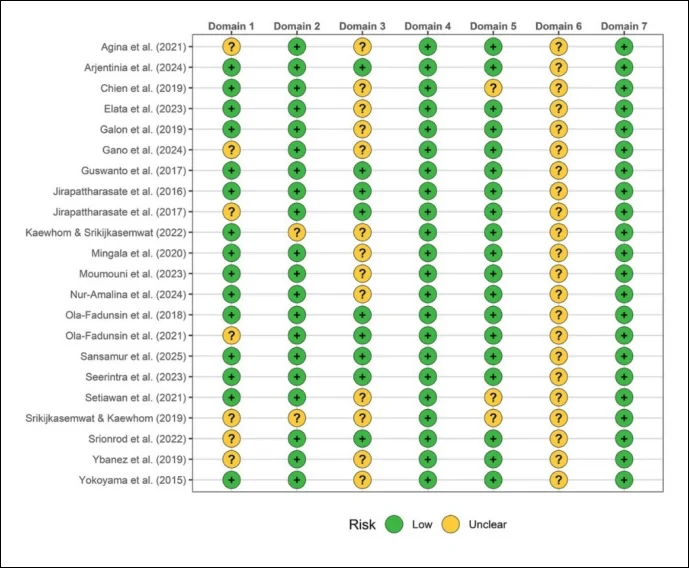
Quality assessment of the included studies. The assessment domains comprised selection bias (Domain 1 = Was the research question clearly described?; Domain 2 = Were the animal characteristics clearly described?), performance bias (Domain 3 = Was the sampling method clearly described?; Domain 4 = Was the polymerase chain reaction-based detection method clearly described?), detection bias (Domain 5 = Were the study subjects categorized into appropriate subgroups?; Domain 6 = Were outcome measurements performed using a blinded method?), and reporting bias (Domain 7 = Was selective reporting bias present?). The maximum quality assessment score was 14 (seven domains). Studies scoring 9–14 were classified as high quality, whereas those scoring 0–8 were classified as low quality. Each domain was scored as 2 (low risk of bias), 1 (unclear risk of bias), or 0 (high risk of bias).

Six studies reported co-infections in cattle, including dual [9, 78–82], triple [9, 79–81], and quadruple infections [81]. The dual co-infection patterns included *A. centrale* with *A. marginale*, *A. marginale* with *B. bigemina*, *A. marginale* with *B. bovis*, *A. marginale* with *T. orientalis*, *A. platys* with *T. sinensis*, *B. bigemina* with *B. bovis*, *B. bigemina* with *T. orientalis*, *B. bovis* with *T. orientalis*, *T. orientalis* with *T. sinensis*, and *T. sinensis* with *Candidatus Mycoplasma haemobos*. Triple co-infections included *A. marginale*, *B. bigemina*, and *B. bovis*; *A. marginale*, *B. bigemina*, and *T. orientalis*; *A. marginale*, *B. bovis*, and *T. orientalis*; *A. marginale*, *T. sinensis*, and *Candidatus Mycoplasma haemobos*; and *B. bigemina*, *B. bovis*, and *T. orientalis*. The only reported quadruple co-infection consisted of *A. marginale*, *B. bigemina*, *B. bovis*, and *T. orientalis*.

### Crude prevalence of hemoparasites in cattle and buffaloes in Southeast Asia

The 22 included studies investigated 5,789 cattle and 901 buffaloes. Hemoparasite infections included *Anaplasma* spp. (932 cattle and 29 buffaloes), *Babesia* spp. (781 cattle and 77 buffaloes), *Theileria* spp. (141 cattle and 40 buffaloes), *Tr. evansi* (128 cattle and 99 buffaloes), *Candidatus M. haemobos* (25 cattle), and *M. wenyonii* (32 cattle and 80 buffaloes).

Among the identified parasite species, *A. centrale* was detected in 23 cattle, *A. marginale* in 868 cattle and 29 buffaloes, and *A. platys* in 41 cattle. Among *Babesia* species, *B. bigemina* was detected in 472 cattle and 33 buffaloes, whereas *B. bovis* was detected in 309 cattle and 44 buffaloes. Among *Theileria* species, *T. orientalis* was detected in 73 cattle and 37 buffaloes, whereas *T. sinensis* was detected in 22 cattle.

The highest reported prevalence of each parasite group was 72.63% for *Anaplasma* spp. in Malaysia [83], 71.13% for *Babesia* spp. in Indonesia [78], 32.79% for *Theileria* spp. in Malaysia [9], 35.45% for *Tr. evansi* in Thailand [93], and 12.58% for *Tr. theileri* in the Philippines [91]. *Candidatus M. haemobos* was reported only in Malaysia, with a prevalence of 40.99% [9], whereas *M. wenyonii* was reported only in the Philippines, with a prevalence of 80.00% [94].

### Crude prevalence of hemoparasites in beef and dairy cattle

*Anaplasma* spp. infections in dairy cattle were reported in three studies [75, 81, 82], with the highest prevalence (31.40%) reported in Malaysia. In beef cattle, *Anaplasma* spp. infections were reported in two studies [9, 80], with the highest prevalence (26.23%) also reported in Malaysia.

*Babesia* spp. infections in dairy cattle were reported in two studies [81, 87], with the highest prevalence (8.39%) reported in Thailand. In beef cattle, *Babesia* spp. infections were reported in five studies [77, 79, 80, 84, 95], with the highest prevalence (37.14%) reported in Malaysia.

*Theileria* spp. infections in dairy cattle were reported in one study from Thailand [81], with a prevalence of 23.40%. In beef cattle, *Theileria* spp. infections were reported in five studies [9, 79, 80, 91, 92], with the highest prevalence (72.13%) reported in Malaysia.

*Tr. evansi* infection was reported exclusively in beef cattle in four studies [9, 86, 91, 93], with the highest prevalence (36.65%) reported in Thailand.

### Target genes for PCR detection of hemoparasites

The PCR target genes identified for each hemoparasite are summarized as follows. *A. centrale* was detected using the 16S ribosomal ribonucleic acid (*16S rRNA*) gene [82]. *A. marginale* was detected using the 16S rRNA, *groEL*, and major surface protein 4 (*MSP4*) genes [9, 75, 76, 80–83, 85, 88]. *A. platys* was detected using the *16S rRNA* gene [9].

For *Babesia* spp., *B. bigemina* was detected using the rhoptry-associated protein 1a (*RAP-1a*) and apical membrane antigen 1 (*AMA-1*) genes [77–81, 84, 87, 88, 90], whereas *B. bovis* was detected using spherical body protein 2 (*SBP-2*), spherical body protein 4 (*SBP-4*), and merozoite surface antigen (*MSA*) genes [77–81, 87, 88, 90, 95].

Among *Theileria* spp., *T. orientalis* and *T. sinensis* were detected using the major piroplasm surface protein (*MPSP*) gene [9, 79–81, 88, 90].

For *Tr. evansi*, the target genes included *RoTat* 1.2 variant surface glycoprotein (*VSG*) [9, 89], internal transcribed spacer (*ITS*) 1 [91], *ITS2* [86], and satellite DNA [93]. *Tr. theileri* was detected using *ITS1* [91]. *Candidatus M. haemobos* and *M. wenyonii* were detected using the 16S rRNA gene [9, 94].

This systematic review provides the first comprehensive, region-wide inventory of molecular diagnostic targets for major hemoparasites infecting cattle and buffaloes in Southeast Asia. The identified molecular targets included 16S rRNA, *groEL*, *MSP4*, *RAP-1a*, *AMA-1*, *SBP-2*, *SBP-4*, *MSA*, *MPSP*, *RoTat* 1.2 *VSG*, *ITS1*, *ITS2*, and satellite DNA. Collectively, these findings represent the first integrated regional synthesis of molecular diagnostic targets for these pathogens and the first comprehensive summary of molecular diagnostic evidence across multiple hemoparasite genera infecting both cattle and buffaloes.

### Pooled prevalence of hemoparasites in cattle and buffaloes in Southeast Asia

The pooled prevalence estimates for the major hemoparasites are presented in Figure 3. *Anaplasma* spp. had a pooled prevalence of 12% (95% CI: 3%-38%; nine studies), with a 95% prediction interval (PI) of 0.37%-89.50% and substantial heterogeneity (I² = 98.6%). *Babesia* spp. had a pooled prevalence of 18% (95% CI: 11%-28%; ten studies), with a 95% PI of 3.16%-61.30% and substantial heterogeneity (I² = 97.0%). *Theileria* spp. had a pooled prevalence of 11% (95% CI: 3%-35%; seven studies), with a 95% PI of 0.43%-84.85% and substantial heterogeneity (I² = 94.8%).

Meta-analysis was not performed for *Candidatus M. haemobos* or *M. wenyonii* because fewer than three eligible studies were available. Species-specific analyses showed that *Tr. evansi* (Figure 3) had a pooled prevalence of 11% (95% CI: 4%-28%; five studies), with a 95% PI of 0.72%-70.64% and substantial heterogeneity (I² = 95.1%). *A. marginale* (Figure 4) had a pooled prevalence of 5% (95% CI: 1%-21%; nine studies), with a 95% PI of 0.29%-72.55% and substantial heterogeneity (I² = 94.7%). *B. bigemina* (Figure 4) had a pooled prevalence of 10% (95% CI: 5%-18%; nine studies), with a 95% PI of 0.88%-54.90% and substantial heterogeneity (I² = 94.6%). *B. bovis* (Figure 4) had a pooled prevalence of 9% (95% CI: 4%-17%; nine studies), with a 95% PI of 0.95%-52.07% and substantial heterogeneity (I² = 97.9%). *T. orientalis* (Figure 4) had a pooled prevalence of 10% (95% CI: 3%-31%; six studies), with a 95% PI of 0.77%-73.70% and substantial heterogeneity (I² = 90.2%).

### Meta-regression and publication bias

The I² values for all pooled analyses exceeded 50% (Figures 3 and 4), indicating substantial between-study heterogeneity. Meta-regression analysis explored potential sources of heterogeneity and showed that the prevalence of *A. marginale*, *B. bigemina*, *T. orientalis*, and *Tr. evansi* was not significantly influenced by geographical region or host species (Table 1). In contrast, the prevalence of *B. bovis* differed significantly between mainland and maritime Southeast Asian countries (meta-regression, p = 0.0048), with a higher pooled prevalence observed in maritime countries.

Visual inspection of the funnel plot (Figure 5) suggested slight asymmetry, indicating possible publication bias. However, Egger's regression test did not detect statistically significant publication bias (p = 0.1293), suggesting no strong evidence of small-study effects. These findings should be interpreted with caution because of the limited number of included studies. Funnel plot assessment and Egger's regression test were performed only for *Babesia* spp., as fewer than ten studies were available for the remaining hemoparasite groups.

**Figure 3:**
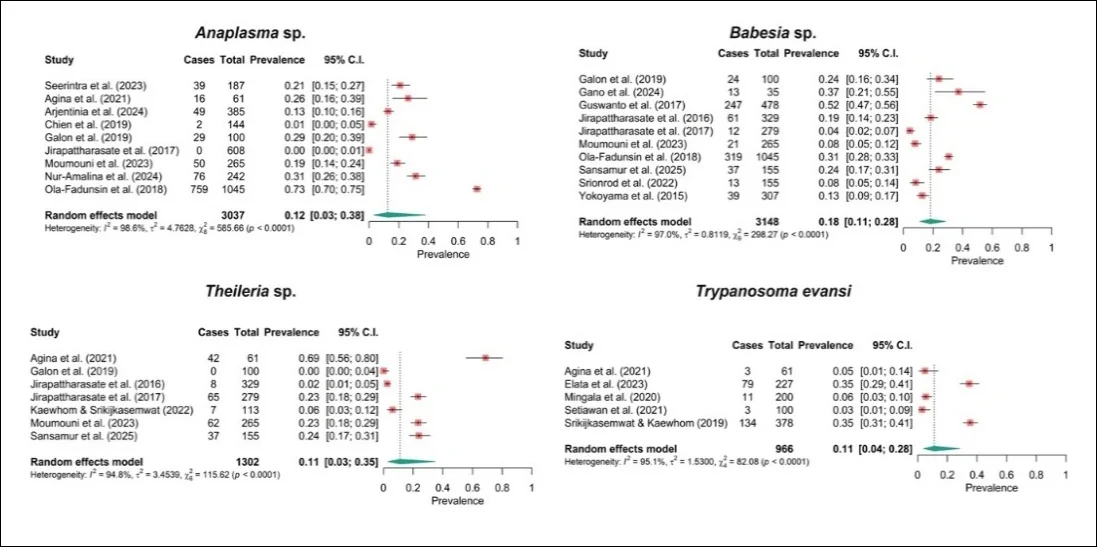
Forest plots showing the pooled prevalence of infections caused by *Anaplasma* spp., *Babesia* spp., *Theileria* spp., and *Trypanosoma evansi* in cattle and buffaloes in Southeast Asia. CI = Confidence interval; PI = Prediction interval.

**Figure 4:**
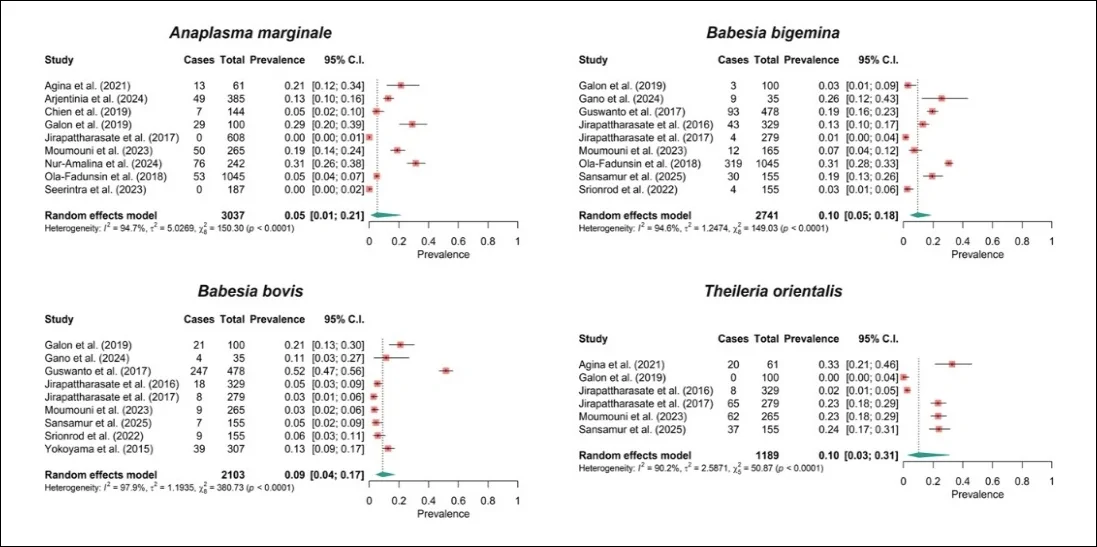
Forest plots showing the pooled prevalence of *Anaplasma marginale*, *Babesia bigemina*, *Babesia bovis*, and *Theileria orientalis* infections in cattle and buffaloes in Southeast Asia. CI = Confidence interval; PI = Prediction interval.

**Figure 5:**
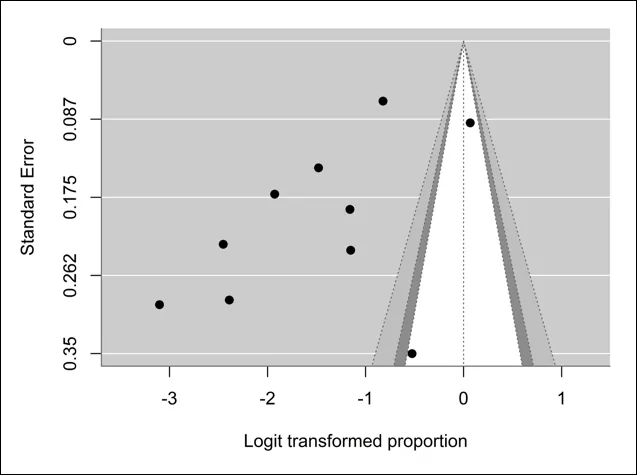
Funnel plot showing publication bias among studies reporting the prevalence of *Babesia* spp. infections in cattle and buffaloes in Southeast Asia.

**Table 1:** Pooled prevalence, heterogeneity, and meta-regression of major hemoparasites in cattle and buffaloes in Southeast Asia.

**Variable**	**Category**	**No. of studies**	**No. examined**	**No. positive**	**Pooled prevalence ** **(95% CI)**	**Q (χ²)**	**p-value**	**I² (%)**	**Meta-regression (p-value)**
*A. marginale*	Region								
	Mainland	8	2,937	931	6.49% (1.93–19.70%)	625.95	<0.001	98.90	0.5522
	Maritime	1	100	29	29.00% (20.11–37.89%)	NA	NA	NA	
	Host species								
	Cattle	8	2,937	931	6.49% (1.93–19.70%)	625.95	<0.001	98.90	0.5522
	Buffaloes	1	100	29	29.00% (20.11–37.89%)	NA	NA	NA	
*B. * *bigemina*	Region								
	Mainland	7	2,263	409	9.55% (3.87–21.68%)	149.50	<0.001	96.00	0.9222
	Maritime	2	578	96	16.61% (13.58–19.64%)	NA	NA	NA	
	Host species								
	Cattle	7	2,586	484	9.57% (3.88–21.72%)	151.81	<0.001	96.00	0.9112
	Buffaloes	2	255	33	12.94% (8.82–17.06%)	NA	NA	NA	
*B. * *bovis*	Region								
	Mainland	7	1,525	94	9.55% (3.87–21.68%)	54.44	<0.001	87.10	0.0048*
	Maritime	2	578	268	46.37% (42.30–50.43%)	NA	NA	NA	
	Host species								
	Cattle	7	1,799	318	9.57% (3.88–21.72%)	358.68	<0.001	98.30	0.4133
	Buffaloes	3	304	44	15.72% (4.65–41.64%)	23.06	<0.001	91.30	
*T. orientalis*	Region								
	Mainland	5	1,089	192	17.31% (7.14–36.29%)	50.87	<0.001	92.10	0.0463
	Maritime	1	100	0	0.00% (0.00–0.00%)	NA	NA	NA	
	Host species								
	Cattle	4	934	155	15.79% (4.93–40.40%)	50.42	<0.001	94.00	0.5197
	Buffaloes	2	255	37	14.51% (10.19–18.83%)	NA	NA	NA	
*Tr. * *evansi*	Region								
	Mainland	2	439	137	31.21% (26.87–35.54%)	NA	NA	NA	0.6143
	Maritime	4	527	93	13.35% (3.64–38.58%)	61.44	<0.001	95.10	
	Host species								
	Cattle	4	518	131	13.24% (3.52–38.94%)	38.48	<0.001	92.20	0.6194
	Buffaloes	3	448	91	20.61% (5.80–52.25%)	45.26	<0.001	95.60	

CI = Confidence interval; χ² = Cochran's Q statistic; I² = Percentage of total variation attributable to between-study heterogeneity; NA = Not available because fewer than three studies were included in the subgroup analysis; *A. marginale* = *Anaplasma marginale*; *B. bigemina* = *Babesia bigemina*; *B. bovis* = *Babesia bovis*; *T. orientalis* = *Theileria orientalis*; *Tr. evansi* = *Trypanosoma evansi*; *p* < 0.05 indicates a statistically significant meta-regression result.

## DISCUSSION

### Prevalence and environmental factors

This systematic review advances the understanding of hemoparasite epidemiology in Southeast Asia in four important ways. First, it provides the first PCR-based pooled prevalence estimates of multiple hemoparasite genera infecting cattle and buffaloes across Southeast Asia during 2015–2025. Second, it presents the first comprehensive inventory of molecular diagnostic targets used in regional studies. Third, it identifies a significant difference in *B. bovis* prevalence between mainland and maritime Southeast Asian countries. Finally, it translates these findings into practical recommendations to strengthen regional veterinary diagnostic capacity, extending the evidence beyond previous single-country reviews.

The pooled prevalence estimates were 12% for *Anaplasma* spp., 18% for *Babesia* spp., 11% for *Theileria* spp., and 11% for *Tr.* *evansi.* These hemoparasites adversely affect livestock health and negatively impact the livelihoods of resource-poor farming communities in developing countries [96]. The tropical climate of Southeast Asian countries, characterized by high temperatures and humidity [97], provides favorable conditions for arthropod vector development [7]. In addition, the region harbors high arthropod vector diversity [8, 15], including 19, 61, 11, 48, 62, and 65 tick species reported from Cambodia, Indonesia, Laos, Malaysia, Thailand, and Vietnam, respectively [7]. Thailand alone has reported 128 tabanid species and 14 *Stomoxyinae* species, which serve as important vectors of hemoparasitic diseases [15]. Collectively, these environmental and ecological factors emphasize the continued risk of vector-borne diseases throughout Southeast Asian countries.

Meta-regression analysis further demonstrated a significant difference in *B. bovis* prevalence between mainland and maritime Southeast Asian countries (p = 0.0048). The higher prevalence observed in maritime countries may reflect ecological conditions that favor tick survival and *B. bovis transmission*. Differences in cattle movement and livestock trade may also contribute to the observed regional variation. However, the interpretation of this finding is limited by the availability of data on vector abundance, environmental conditions, livestock movement, and management practices across the two regions. These knowledge gaps highlight the importance of adopting a One Health approach, together with consideration of socioeconomic factors, to improve the understanding, surveillance, and control of hemoparasitic and other zoonotic diseases in Southeast Asian countries.

### Co-infection patterns, economic impacts, and zoonotic potential

The synthesized evidence demonstrated diverse hemoparasite co-infection patterns in both cattle and buffaloes. Cattle exhibited dual, triple, and quadruple co-infections, whereas buffaloes exhibited only dual and triple co-infections. Co-infections involving multiple hemoparasites are likely to result in more severe clinical manifestations than single-species infections because of the combined pathogenic effects of different parasites. For example, *A. marginale* induces anemia through splenic clearance of infected erythrocytes, whereas *Babesia* spp. directly destroy erythrocytes during intracellular replication [10, 11]. Concurrent infection with these pathogens may therefore contribute to more severe disease.

Previous studies have reported substantial production losses associated with *Anaplasma*, *Babesia*, and *Theileria* infections, resulting in considerable economic losses due to reduced productivity and increased mortality [98]. In addition, *A. platys* and *Tr. evansi* have been reported in human infections, suggesting potential zoonotic importance, although their zoonotic roles remain to be fully established [14, 99]. The findings of the present study provide baseline information to support future epidemiological and molecular investigations of transmission pathways, zoonotic potential, vector control, acaricide resistance, and the effects of climate change on hemoparasite distribution. A One Health approach will be essential to strengthen surveillance systems and improve the prevention and control of these diseases in Southeast Asia.

### Diagnostic techniques for hemoparasites in cattle and buffaloes

The diagnosis of hemoparasite infections has traditionally relied on clinical signs together with laboratory detection of the causative agents [96]. PCR has been demonstrated to be a sensitive and reliable method for the molecular detection of hemoparasites in animals [100]. This systematic review provides the first comprehensive, region-wide synthesis of molecular diagnostic targets for the major hemoparasites infecting cattle and buffaloes in Southeast Asia by integrating evidence across multiple parasite genera. The compiled molecular targets may serve as a useful reference for veterinary diagnostic laboratories and help improve laboratory diagnostic capacity and technical competency.

However, PCR-based assays may not be readily available in all settings because of financial and technical constraints. In such circumstances, conventional methods, including blood smear and buffy coat smear examinations, remain practical and cost-effective diagnostic alternatives [101], particularly in rural areas where cattle and buffaloes are raised as sources of household income and financial security. The molecular and parasitological information summarized in this review may also help veterinary technicians and laboratory personnel improve their diagnostic competency for hemoparasitic diseases.

### Limitations of the systematic review and meta-analysis

This systematic review and meta-analysis has several limitations. First, substantial between-study heterogeneity was observed, with I² values of 98.60%, 97.00%, 94.80%, and 95.10% for *Anaplasma*, *Babesia*, *Theileria*, and *Trypanosoma*, respectively. This heterogeneity may reflect differences in geographical location, study populations, host species, sample size, and study methodology. Although meta-regression identified geographical region and host species as significant contributors to heterogeneity for *Babesia* infections, a considerable proportion remained unexplained.

Second, although visual inspection of the funnel plot suggested possible publication bias for *Babesia* studies, Egger's regression test did not provide statistically significant evidence of publication bias. Moreover, these analyses could not be performed for the remaining hemoparasites because fewer than ten studies were available for each group, limiting the ability to assess potential publication bias and the possibility of underestimated pooled prevalence estimates.

Finally, the inclusion criteria restricted the analysis to PCR-based studies published in the English language and excluded studies using serological methods. These selection criteria may have introduced publication and language biases and should be considered when interpreting the findings of this review.

## CONCLUSION

This systematic review and meta-analysis provides the first comprehensive synthesis of PCR-confirmed prevalence and molecular diagnostic targets of major hemoparasites infecting cattle and buffaloes across Southeast Asia during 2015–2025. Analysis of 22 cross-sectional studies demonstrated pooled prevalences of 12% for *Anaplasma* spp., 18% for *Babesia* spp., 11% for *Theileria* spp., and 11% for *Tr*. *evansi*, indicating that these pathogens remain widely distributed throughout the region. The study further identified a significantly higher prevalence of *Babesia bovis* in maritime than mainland Southeast Asian countries and compiled the principal molecular targets currently used for PCR-based diagnosis, including 16S rRNA, *groEL*, *MSP4*, *RAP-1a*, *AMA-1*, *SBP-2*, *SBP-4*, *MSA*, *MPSP*, *RoTat* 1.2 *VSG*, *ITS1*, *ITS2*, and satellite DNA.

The findings have important practical implications for veterinary diagnostics and disease surveillance. The consolidated molecular diagnostic targets provide a valuable reference for selecting appropriate PCR assays and may help harmonize molecular diagnostic protocols among veterinary laboratories in Southeast Asia. At the same time, conventional diagnostic approaches, such as blood smear and buffy coat smear examinations, remain practical and cost-effective alternatives for routine screening in resource-limited settings where molecular techniques are unavailable.

A major strength of this study is that it represents the first PRISMA-guided, region-wide meta-analysis integrating PCR-confirmed prevalence estimates, molecular diagnostic targets, co-infection patterns, and meta-regression analyses across multiple hemoparasite genera in cattle and buffaloes.

Future research should expand molecular surveillance to underrepresented Southeast Asian countries, standardize molecular diagnostic methodologies, integrate vector ecology and livestock movement data with molecular epidemiology, and investigate the influence of environmental and management factors on hemoparasite transmission using a One Health framework. Longitudinal studies and genomic surveillance will further improve understanding of pathogen diversity, transmission dynamics, and emerging epidemiological trends.

In conclusion, this study provides robust regional evidence on the molecular epidemiology of major bovine hemoparasites in Southeast Asia and establishes a comprehensive reference for molecular diagnosis and disease surveillance. The findings contribute to strengthening veterinary diagnostic capacity, supporting evidence-based surveillance programs, and improving the prevention and control of hemoparasitic diseases in cattle and buffaloes, thereby enhancing livestock productivity, food security, and the sustainability of the livestock sector across the Southeast Asian countries.

## DATA AVAILABILITY

The data generated during the study are included in the manuscript.

## GENERATIVE AI DECLARATION

The authors declare that no generative AI was used in the preparation of this manuscript.

## AUTHORS’ CONTRIBUTIONS

NP, NS, and PP: Conceptualization, methodology, data collection, and data curation. NP, NS, TNT, and PP: Investigation and formal analysis. PP: Software and validation. NS: Writing—original draft. NP, TNT, and PP: Writing—review and editing. All authors have read and approved the final manuscript.
